# Comments: Effect of the location and size of thyroid nodules on the diagnostic performance of ultrasound elastography: A retrospective analysis

**DOI:** 10.6061/clinics/2021/e2891

**Published:** 2021-05-27

**Authors:** Demet Sengul, Ilker Sengul

**Affiliations:** I Department of Pathology, Giresun University Faculty of Medicine, 28100 Giresun, Turkey.; II Division of Endocrine Surgery, Giresun University Faculty of Medicine, 28100 Giresun, Turkey.; III Department of General Surgery, Giresun University Faculty of Medicine, 28100 Giresun, Turkey.

We read with great interest the research article entitled “Effect of the location and size of thyroid nodules on the diagnostic performance of ultrasound elastography: A retrospective analysis” by Xie and Yu (1). The authors claimed that the results of strain ultrasound elastography should be carefully evaluated when small thyroid nodules are close to the carotid artery. They indicated that the use of cytology to determine nodule parameters is effective in determining the diagnostic ability of strain ultrasound elastography. However, they discussed only benign and suspicious nodules throughout their article.

According to the Bethesda System for Reporting Thyroid Cytopathology (TBSRTC), 1st ed., the risk of malignancy (ROM) from indeterminate cytology is as follows: III, atypia of undetermined significance or follicular lesion of undetermined significance (AUS/FLUS), 5%−15%; IV, follicular neoplasm (FN) or suspicious for a follicular neoplasm (SFN), 15%−30%; and V, suspicious for malignancy (SM), 60%−75%, while in the TBSRTC 2nd ed., the ROMs are 10%−30%, 25%−40%, and 50%−75%, respectively.

The leading thyroid cytology classification systems have categorized indeterminate cytology and their associated ROMs into categories equivalent to III, IV, and V of the TBSRTC.

The current 2016 UK Royal College of Pathologists (RCPath) Thy terminology divides the indeterminate cytology category Thy3 into Thy3a, neoplasm possible, atypia/nondiagnostic, and Thy3f, neoplasm possible, suggestive of FN, with ROMs of 20%−31%, and 24%−39%, respectively, and defines Thy4 as SM with a ROM of 70%−87%.

The 2014 Italian Consensus for the Classification and Reporting of Thyroid Cytology (ICCRTC) divided its diagnostic category TIR3, indeterminate cytology, into two subcategories: TIR3A (low-risk indeterminate lesion, LRIL) and TIR3B (high-risk indeterminate lesion, HRIL), with different expected cancer risks and discrete clinical characteristics. TIR3A is characterized by augmented cellularity with numerous microfollicular structures in a low colloid background or a scarce cellular structure consisting predominantly of microfollicular groups with oxyphilic features (Hurthle cells), with an expected cancer risk of <10%. TIR3B is characterized by mild/focal nuclear atypia, with an expected cancer risk of 20%−30%. The ICCRTC also includes category TIR4, SM with an expected malignancy risk of 60%−80%.

The 2014 Royal College of Pathologists of Australasia (RCPA) and Australian Society of Cytology (ASC) categorized thyroid nodules as follows: 3, indeterminate (AUS/FLUS) with a low ROM of 5%−13%; 4, suggestive of an FN (FN/SFN) with a moderate ROM of 21%−26%; and 5, SM with a high ROM of 85%−90%.

The 2013 Japan Thyroid Association (JTA) Guidelines for the Management of Thyroid Nodules categories are as follows: indeterminate B, other tumors, (TBSTRC III); indeterminate A, FN, (TBSRTC IV), i) indeterminate A1, favor benign, ii) indeterminate A2, borderline, iii) indeterminate A3, favor malignant; and SM (TBSRTC V).

In addition, the first one remains a crucial challenging cytological category in all systems. Category I in TBSRTC, 1st and 2nd ed., is Nondiagnostic or Unsatisfactory. Category Thy1 in the 2016 RCPath is Nondiagnostic for cytological diagnosis and Thy1c is Nondiagnostic for cytological diagnosis-cystic lesion. Category TIR1 in the 2014 ICCRTC/Italian Society for Anatomic Pathology and Cytology−Italian Thyroid Association (SIAPEC-AIT2013) is Nondiagnostic and TIR1c is Nondiagnostic cystic. Category 1 in the 2014 RCPA/ASC is Nondiagnostic, and Category 1 in the 2013 JTA is Inadequate. These categories are still used in the management of suspicious (not just indeterminate cytology) nodules (2-7). Therefore, a suspicious nodule may be classified as AUS/FLUS, FN/SFN, SM, and even as nondiagnostic, all of which have a wide range of ROMs. Is it important to point out the cytological categories of the thyroid nodules in the research by Xie and Yu? Has designating the nodules as “suspicious”, while ICCRTC divided TIR3, indeterminate cytology, into A, LRIL and B, HRIL (even though the latter corresponds to TBSRTC IV unlike the former) and even sub-typing TBSRTC III is being discussed, nowadays, by some authors, affected their results? This issue merits further investigation. We thank Xie and Yu for their valuable study.
